# Strategies to enhance the ability of nerve guidance conduits to promote directional nerve growth

**DOI:** 10.1186/s12938-024-01233-z

**Published:** 2024-04-06

**Authors:** Ziyue Zhang, Muyuan Ma

**Affiliations:** South China University of Technology School of Medicine, Guangzhou, China

**Keywords:** Nerve guidance conduits, Directional structures, Contact guidance, Electrical stimulation, Concentration gradient

## Abstract

Severely damaged peripheral nerves will regenerate incompletely due to lack of directionality in their regeneration, leading to loss of nerve function. To address this problem, various nerve guidance conduits (NGCs) have been developed to provide guidance for nerve repair. However, their clinical application is still limited, mainly because its effect in promoting nerve repair is not as good as autologous nerve transplantation. Therefore, it is necessary to enhance the ability of NGCs to promote directional nerve growth. Strategies include preparing various directional structures on NGCs to provide contact guidance, and loading various substances on them to provide electrical stimulation or neurotrophic factor concentration gradient to provide directional physical or biological signals.

## Introduction

Traffic accidents, natural disasters and other trauma often result in peripheral nerve injury (PNI). According to epidemiological studies published in 2018, the average annual incidence rates of PNI in the upper and lower limbs in the United States were 43.8/1 million people and 13.3/1 million people respectively, which can lead to lifelong disability and a heavy financial burden [1, 2].

Sydney Sunderland classified nerve injuries into five degrees, and the higher degrees indicate more severe damage and greater loss of motor, sensory and autonomic functions. It is generally believed that injuries of third degree and above are not completely reversible, and require surgical intervention to reduce permanent functional impairment. The main causes of incomplete nerve repair are erroneous cross-shunting and loss of axons [3, 4].

Observation studies based on electron microscopy have confirmed that after nerve dissection injury, the proximal nerve fibers send out multiple side branches and terminal buds. The buds serve as "regenerative units" and grow to the distal nerve segments, which can repair the damage [5]. If lots of the buds fail to reach the correct distal connection site, they will be pruned and removed, which may delay axon regeneration [6]. This is because the side branches that lack direction of growth compete for too much structural material, resulting in insufficient material for the dominant buds that grow in the relatively correct direction, which makes the regeneration process slow and incomplete [7]. Besides, loss of neurotrophic support to target organs, fibrin deposition and long-term denervation of Schwann cells (SCs) are also not conducive to axon regeneration [8, 9].

Thus, guiding axons of injured nerves to regenerate in the correct direction may speed up the repair process of nerves and promote the recovery of their functions. Based on this, various NGCs have been developed and fabricated to provide temporary structure and directional guidance for migration of SCs and axonal growth, and are eventually replaced by regenerative cells and extracellular matrix [10]. Several NGCs have been approved for clinical use to connect nerve defects within 3 cm, such as Avance® Nerve Graft and AxoGuard™ Nerve Connector developed by AxoGen Inc., most of which are composed of acellular biological tissues such as acellular human nerve allograft [11]. However, the clinical application of NGCs is still limited, mainly because it is difficult for them to imitate the complex anatomical structure and regeneration microenvironment of natural peripheral nerves, so their effect in promoting nerve repair is not as good as autologous transplantation [12].

There exist many reasons why autologous transplantation can offer better directional cues compared with NGCs. Firstly, using autografts to repair nerve defects rarely lead to tissue rejection, while the materials of NGCs are usually not so biocompatible and may even inhibit cell proliferation [13]. In addition, by interfascicular dissection, the nerve autografts can be split into fascicles, which can be connected individually with the fascicles of the injured nerves during the surgery. Therefore, compared with NGCs, autologous transplantation can achieve a more precise coaptation, promoting the axons to extend in the correct direction [14]. In addition, autografts used to repair the defects should contain capillaries, fibroblasts and a large quantity of SCs [15]. These components inherently within the autografts participate in forming a microenvironment conducive to nerve regeneration. However, when NGCs are used to repair the defects, only after the process of angiogenesis and cell adhesion completes can a similar microenvironment be formed within them. In some studies, SCs have been seeded within the conduits [9], but their ability to promote nerve regeneration may also be inferior to the ones in autografts. This is because SCs need to express various phenotypes so as to produce a variety of neurotrophic factors when regulating the regeneration of different nerves [16]. Most SCs within autografts may have already expressed the appropriate phenotype, while the ones seeded within NGCs may still need to undergo phenotypic transition. Thus, autologous transplantation is still the gold standard to bridge nerve defects, and there has been meta-analysis suggesting that its meaningful recovery rate after operation is indeed significantly better than that of conduit repair [17].

**Fig. 1:**
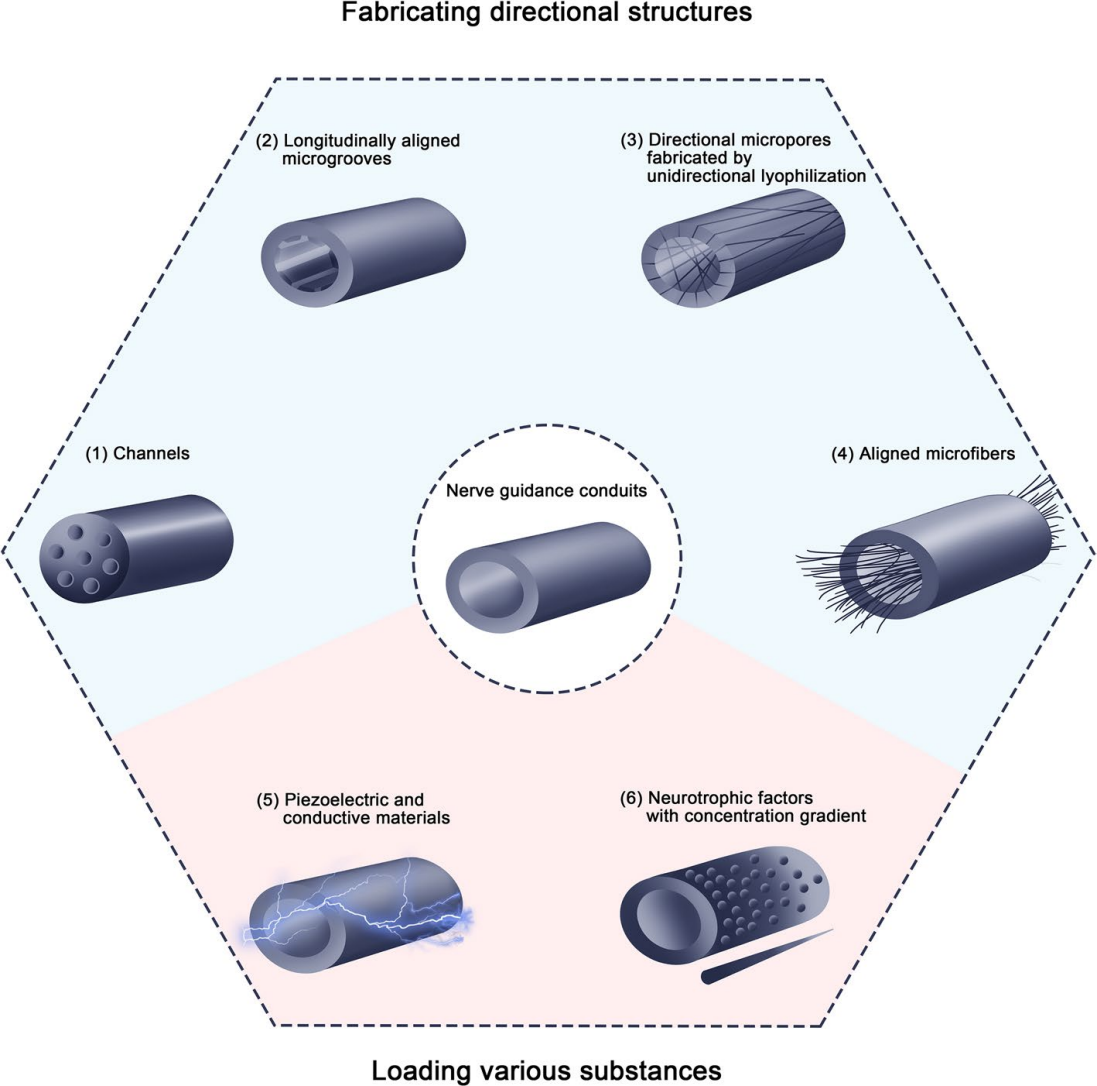
Schematic representation of main strategies to enhance the ability of NGCs to promote the directional growth of nerves. Depicted strategies include fabricating directional structures and loading various substances within NGCs

To make the effect of NGCs in promoting nerve repair closer to that of autologous transplantation, there exist many measures that can be taken to improve the conduits. For example, appropriately sized micropores can be prepared within the conduits to ensure adequate supply of oxygen and nutrients to maintain the activity of SCs, which nerve repair is highly dependent on [18, 19]. In addition, by improving the materials used to prepare NGCs, the tissue compatibility as well as degradability of the conduits can be enhanced, which reduces tissue rejection and further promote nerve regeneration [20]. The improvement strategies mainly introduced below are to promote the oriented growth of nerve fibers by further enhancing the directional guidance ability of NGCs. The main improvement strategies include fabricating directional structures in the conduits and loading various substances within them (Fig. 1). To test the ability of NGCs to promote nerve regeneration, many methods have been used in the studies (Table 1).

**Table 1:** Methods to test the ability of NGCs to promote nerve regeneration

Experiment types	Methods	References
Cell culture in vitro	Cell morphology observation	[21, 22, 23, 24, 25, 26]
	Detection of differentiation-related substances	[27, 28, 29, 30]
	Detection of myelination-related substances	[31]
Repair of rat sciatic nerve defects in vivo	Histologic section observation	[12, 32, 33]
	Immunofluorescence staining	[29, 34]
	T2 signal changes in MRI of injured nerves	[35]
	Electrophysiological signal detection	[12, 33, 34]
	Reflex function measurement	[12, 33]
	Motor function measurement	[12, 33, 34]
	Atrophic gastrocnemius muscle weighing	[12, 33]

## Fabricating directional structures in the NGCs

### The mechanisms of structures promoting directional growth

The directional structures of the conduits can provide geometry cues and contact guidance, affecting the direction of cell elongation and the polarization of intracellular structures during the nerve regeneration process. Contact guidance means that when confronted with a unidirectional guidance structure, cells will be forced to grow along a single course. This phenomenon was first proposed by Paul Weiss when describing cell growth specificity [36].

**Fig. 2:**
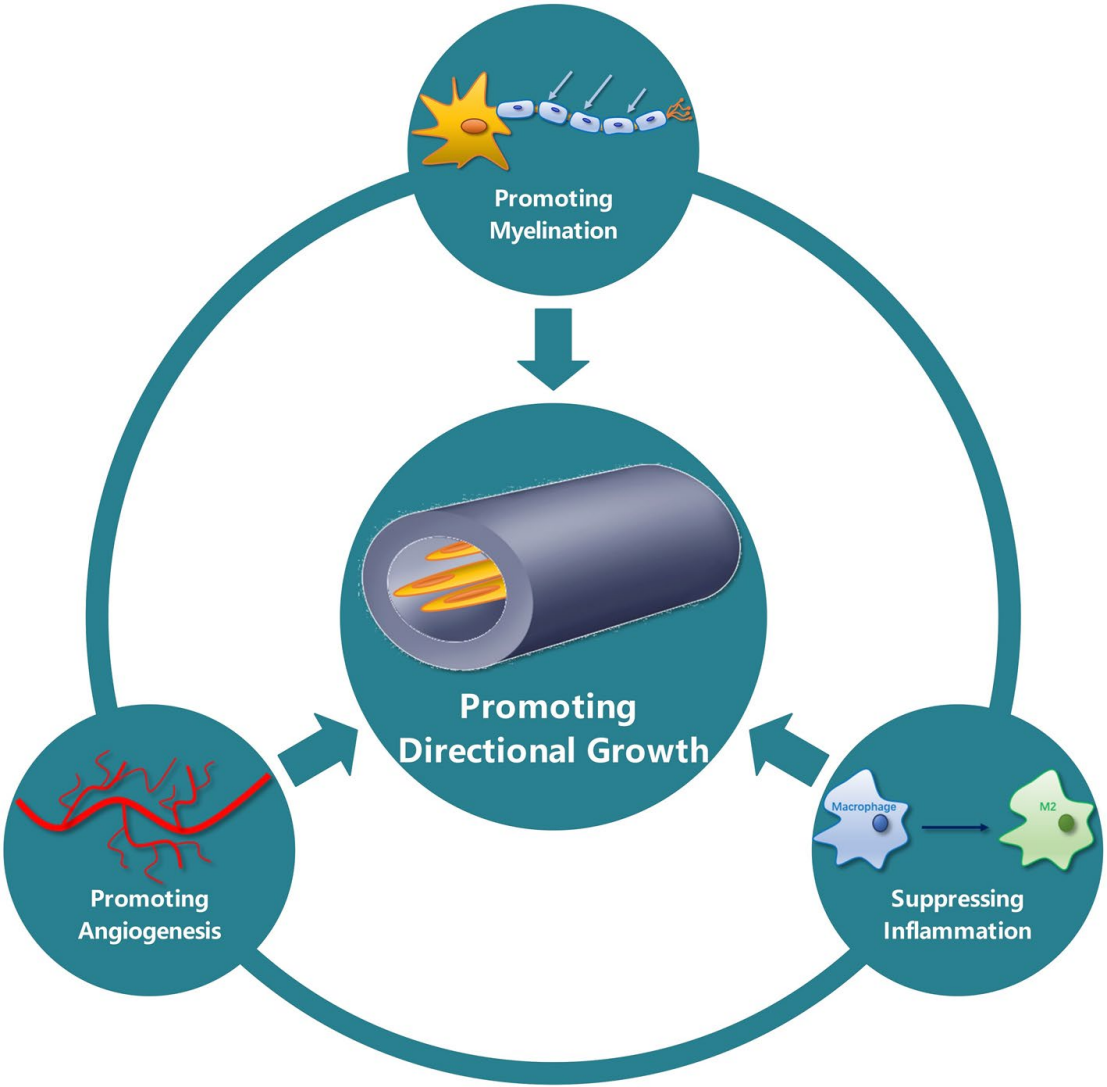
Schematic representation of mechanisms of structures promoting directional growth. Depicted mechanisms include promoting myelination and angiogenesis, and suppressing inflammation

It was found that when cells were cultured on materials with randomly oriented microstructures, the cells overall appeared polygonal, and their growth was randomly oriented as well. In contrast, when the cells grew on materials with oriented structures, they aligned and elongated along the axis of the structures, and the actin cytoskeleton and nucleus within them also responded by becoming polarized [28]. Contact guidance can also promote the differentiation of stem cells into neurons, causing them to exhibit typical characteristics of neuronal cells, such as multipolar elongation and expression of neurofilament proteins [27]. This is related to the influence of conduit surface topographies on gene expression levels of the cells [37]. Many previous studies have shown that the directional structures in conduits such as channels, groove geometry, directional micropores fabricated by unidirectional lyophilization, and microfibers can effectively promote nerve regeneration (Fig. 2) [38].

### Various structures fabricated in NGCs

#### Channels

Researchers prefer to prepare multi-channel structures in the NGCs rather than traditional single-channel hollow ones. Because the former can make the structures of NGCs closer to that of the natural peripheral nerve bundle, being more conducive to material exchange and cell adhesion, providing templates for axon elongation, and promoting angiogenesis during nerve regeneration [12, 39]. The main method to prepare multi-channel structures is to change the shape of the mold. For example, metal wires can be inserted into the mold and used as mandrels [32, 40]. Some researchers choose to roll up the fiber sheets made by electrospinning on the rod to make single-channel small conduits, and then combine several small conduits into a multi-channel one [41, 42]. Shape memory materials, which can be temporarily flattened at room temperature to facilitate the loading of cells, can also be used to create multi-channel NGCs [39].

In vitro cell culture shows that conduits with multi-channel structures can promote adhesion and proliferation of SCs, and promote the differentiation of stem cells. Multi-channel structural NGCs were used to repair sciatic nerve defects in rats, and it was found that the regenerated nerve recovered in many aspects. For example, the regeneration of axons and surrounding vessels, the recovery of reflex function and atrophic gastrocnemius muscle innervated by the sciatic nerve have been observed. As for motor function recovery, it was found to be statistically equivalent to autologous transplantation. If multi-channel conduits are seeded with SCs, their ability to promote axonal regeneration can even be better than autografts [12, 32].

#### Longitudinally aligned microgrooves

Longitudinally aligned microgrooves endowed in NGCs can also further increase the ability of the conduits to promote directional nerve growth by increasing conduit surface area, and providing contact guidance for nerve regeneration with its highly ordered structure. The microgrooves in the conduits are mostly fabricated by pressing and etching a micropatterned mold or stamp on the matrix. The mold used can be prepared by photolithography and 3D printing [21, 22, 43]. The matrix can also undergo a light-triggered free radical reaction on the micropatterned mold, which causes it to rapidly cross-link and gel to form microgrooves [31]. Some researchers formed voids on the spray surface in the initial stage of electro-spinning, then fabricated microgrooves on the electro-spun fiber surface through the elongation and solidification of the voids [34].

When culturing SCs on materials with microgrooves, the structures can guide the directional migration of SCs, make them more evenly distributed [21, 22], and promote them to form myelin [31]. Microgrooves could also promote neural stem cells to differentiate into glial lineages and neurons [43], and contribute to angiogenesis [21]. Using conduits with microgrooves to repair rat nerves resulted in better electrophysiological assessment results and motor function recovery after nerve regeneration. It was found that the microgrooves made the diameter of the regenerated axons and the thickness of the myelin sheath larger, the density of neurofilaments higher, the number of SCs greater and their distribution better [34].

#### Directional micropores fabricated by unidirectional lyophilization

To form a directional microporous structure within the NGCs to provide contact guidance for nerve regeneration, the unidirectional lyophilization method can be used [44]. The method is to place the mold, which contains the solution used to prepare the NGCs, vertically on a metal plate in liquid nitrogen, so that one side of the conduit is in contact with the cooling plate surface, while the other side is at room temperature, forming a temperature gradient vertically. Then, the solution in the mold freeze in specific directions to form ice particles, which act as pore-making agents and create highly interconnected longitudinal porous structures in the conduit after sublimation [29, 45, 46]. Further research found that the size of the micropores could be controlled by changing the concentration of the solution used [47], and the consistency of the distribution and orientation of the formed micropores mainly depends on the rate and temperature of freezing [48]. Some researchers have also tried to further improve the conduits by loading nanotubes in directional micropores to sustainably release drugs [24].

Cell culture was conducted on materials with directional micropores formed by unidirectional lyophilization, and it was found that the microporous structure can effectively guide the migration of SCs, and the cells can infiltrate into the micropores. The oriented micropores can also make growth of axons more directional and increase axonal length [23, 24, 46, 48]. Using the NGCs to repair nerve defects, it was found that longitudinal micropores can promote directional extension of axons and myelination [45].

#### Aligned microfibers

Inserting microfibers into the lumen of NGCs is also a strategy to provide contact guidance for nerve regeneration. Microfibers used for conduit preparation are mostly produced by electro-spinning [25, 27, 35, 39, 46, 49, 50]. Drugs can also be encapsulated in these microfibers to further promote the recovery of injured nerves [28]. Microfibers can also be prepared with the help of melt electro-writing 3D printing technology, which can well control the diameter and distribution of microfibers [29].

In vitro cell culture results show that SCs not only grew on the surface of the conduits, but also infiltrated between the microfibers in the lumen [25]. Compared with randomly distributed microfibers, aligned microfibers can promote the proliferation, migration and myelination of SCs, and directional axonal extension during nerve regeneration [35, 39, 46]. Microfibers can also induce a higher proportion of mesenchymal stem cells to differentiate into neurons and glial cells. [27–29]. Using the NGCs to repair nerve defects, it was found that aligned microfibers were more conducive to migration of SCs and axon elongation, allowing more myelinated nerve fibers to regenerate and distribute well along the conduit long axis. However, the axon density of regenerated nerves repaired by conduits was still not as high as that of the ones repaired by autografts [25, 39, 49, 50]. Some researchers found that microfibers could promote nerve repair by inducing angiogenesis, suppressing inflammation and reducing fibroblast infiltration, which may reveal part of the mechanism by which aligned microfibers promote nerve repair [29].

## Loading various substances within NGCs

### The mechanism of substances promoting directional growth

Loading various substances within NGCs is also one of the commonly used strategies to promote nerve regeneration [51]. To further improve the ability of NGCs to promote directional neural growth, providing directional guidance signals through loaded substances may be a feasible method. Here we mainly describe piezoelectric and conductive materials that can provide directional physical signals, and neurotrophic factors with concentration gradient that can provide directional biological signals.

Some studies have partly explained the way electrical stimulation promotes nerve regeneration at the molecular level, such as affecting the polarization of macrophages and activating the signaling pathway related to angiogenesis. After the occurrence of PNI, the pro-regenerative macrophages (M2) can promote the nerve repair by secreting anti-inflammatory cytokines, while the pro-inflammatory ones (M1) are not conducive to nerve regeneration. Some researchers found that electrical stimulation can regulate the intracellular ion concentration of macrophages, which increases the expression of interleukin-4 receptor subunit alpha (IL-4R*α*) mediating M2 polarization. However, the expression of toll-like receptor 4 (TLR-4) mediating M1 polarization is not influenced by electrical stimulation. Thus, the electro-active materials can promote M2 polarization induced by interleukin-4 (IL-4), playing an immuno-modulatory role in nerve regeneration [52, 53]. Electrical stimulation may also activate the protein kinase B or Akt (PKB/Akt) signaling pathway to accelerate the repair of injured nerves by promoting angiogenesis. It has been found that PKB/Akt can be phosphorylated under electrical stimulation, then the downstream endothelial nitric oxide synthase (eNOS) is activated, which finally increases the production of vascular endothelial growth factor (VEGF). After binding to receptors expressed by endothelial cells, VEGF serves as an important mitogen to further trigger a variety of downstream signals that promote angiogenesis [54–56]. The electrical signals that injured nerves receive is directional, so the occurrence of the molecular mechanisms above may also have a certain degree of directivity.

Neurotrophic factors such as nerve growth factor (NGF) also play an important role in promoting nerve regeneration, and some further studies have revealed the possible molecular mechanisms by which NGF exerts its effects on the injured nerves. For example, Li. et al. found that NGF can bind to the 75 kD neurotrophin receptor (p75^NTR^) of SCs to activate the downstream AMP-activated protein kinase (AMPK), thereby inhibiting the activation of mammalian target of the rapamycin (mTOR), which is a negative regulator of autophagy. Therefore, this p75^NTR^/AMPK/mTOR-dependent pathway can accelerate the degradation of myelin fragments in injured nerves by enhancing the autophagy of SCs, shortening the time required for myelin remodeling during nerve regeneration [57]. And if the neurotrophic factors within NGCs can form a concentration gradient, the activation of the above-mentioned signaling pathways in injured nerves may also be directional.

### Piezoelectric and conductive materials

#### Effects of electrical stimulation on nerve regeneration

Piezoelectric materials and conductive materials loaded in NGCs can promote the growth direction accuracy of injured nerves during their regeneration by generating and conducting electrical stimulation signals.

Shah, M.B. et al. and Ma, Y. et al. each fabricated NGCs with channels. The conduits of former were not electrically active, while the ones of latter were loaded with poly-vinylidene fluoride (PVDF) and poly-(3,4-ethylenedioxythiophene) (PEDOT). Two types of conduits were used to repair nerves in vivo respectively, and it was found that the electro-physiological signals of the nerves repaired by the former could not be detected, while the latency of the collected compound muscle action potentials of the nerves repaired by the latter was equivalent to that of autologous transplantation, indicating that electrical stimulation is beneficial to the recovery of nerve conduction function [12, 33]. Several in vitro cell culture and in vivo nerve repair experiments also found that short-term and low-frequency electrical stimulation could accelerate axonal regeneration [7, 58, 59]. The mechanism by which electrical stimulation promotes nerve regeneration has not been fully elucidated yet, and immune-suppression as well as promoting angiogenesis may be part of it [52–54].

#### Piezoelectric materials

To apply electrical stimulation to injured nerves, piezoelectric materials can be added to the NGCs used to connect the nerve defect. Such materials can generate different surface charges under mechanical triggering, and can effectively and synchronously convert pressure into pulse electrical signals without the need for additional energy sources or electrodes. Since the autonomic nervous system can drive the tissues to provide pressure, the electrical stimulation signal provided by piezoelectric materials can be adjusted by own nerve impulses of the organism, so that the frequency and intensity of the electrical stimulation are synchronized with the physiological state [60]. Among various piezoelectric polymers, PVDF has better piezoelectric properties.

When conducting in vitro cell culture experiments, researchers used mechanical vibration to generate an electric field on the PVDF surface, and found that the generated electrical stimulation can promote the growth of neuron axons [26]. Studies have used NGCs containing PVDF to connect sciatic nerve defects in mice, proving that PVDF can indeed promote the regeneration of peripheral nerves in the organism [61].

#### Conductive materials

To conduct electrical stimulation to the nerve defects, the substances loaded within NGCs also requires a certain degree of conductivity. Conductive materials used for conduit preparation mainly include various organic conductive polymers, graphene and their derivatives.

Conductive polymers include poly-pyrrole (PPy), poly-aniline (PANI) and PEDOT. Among various conductive polymers, PEDOT exhibits better biocompatibility, physical and chemical stability, and electro-chemical properties [62]. Whether PEDOT is added to the matrix of cell culture in vitro or implanted into animals for in vivo testing, it shows no obvious cytotoxicity [63]. Cell culture showed that compared with PPy, using PEDOT as the conductive material in the conduits made neurites appear more slender and have fewer branches, suggesting that this type of polymer could make nerve regeneration more directional [64].

Graphene and its derivatives, like graphene oxide (GO) and reduced graphene oxide (rGO), are also used to prepare electro-active NGCs. The toxicity of graphene is dose-dependent, and the content of such substance in electrically active NGCs is often very low, which is not enough to induce organ damage [29, 65]. Studies found that materials containing graphene derivatives could promote the differentiation of neural stem cells in vitro [43]. After nerve repair surgery, the conduits with such materials can also promote the recovery of motor function and reduce muscle atrophy as well as collagen deposition [66].

### Neurotrophic factors with concentration gradient

It was discovered that neurotrophic factors promote the selective regeneration of axons towards distal nerve stumps and target organs. When nerve injury occurs, the expression level of neurotrophic factors like NGF will be significantly increased in the distal stump. The cells can receive and respond to biological signals provided by differences in NGF concentration in the environment, so their regeneration is directional [20]. Therefore, by loading neurotrophic factors into conduits and forming a concentration gradient, it is possible to further enhance the ability of NGCs to promote directional nerve regeneration.

There exist many ways to create a concentration gradient of neurotrophic factors within NGCs. A concentration gradient can be formed by sequentially injecting nutrient factor solutions of different concentrations into the mold [67]. The conduits can also be placed vertically in a container, into which the neurotrophic factor solution is then injected. The amount of solution absorbed by the upper and lower parts of the conduits is different, which can form a concentration gradient [68]. Some researchers loaded neurotrophic factors into microspheres, and through continuous centrifugation of microspheres loaded with different amounts of nutritional factors, a concentration gradient could be quickly formed [69].

In vitro cell culture showed that NGFs with concentration gradient could promote neurite elongation better than the ones with uniform concentration. Such NGCs were used to repair the injured sciatic nerves, and it was found that the number of myelinated nerve fibers and the thickness of the myelin sheath, the recovery of conduction and motor function of the regenerated nerves were statistically equivalent to those of autografts, and the density of regenerated axons was even higher than autologous transplantation [67, 68]. These findings may indicate that the concentration gradient of neurotrophic factors in NGCs is beneficial to nerve regeneration.

## Conclusion and future perspectives

### Summary of the introduced improvement strategies

To reduce the loss of function caused by the non-directional extension of axons during nerve regeneration, various NGCs have been fabricated to provide directional guidance for nerve repair. In some studies, the conduits developed could be statistically equivalent to autologous transplants in some aspects, such as promoting the recovery of motor function, and could even be better than autografts in terms of increasing the density of regenerated axons. However, the advantages of these conduits were often limited to a few aspects [12, 32, 49, 68]. Overall, most conduits developed are still not as good as autografts in promoting nerve repair.

Improving the structure of the conduits to provide contact guidance for nerve regeneration, loading various substances within the conduits to provide directional physical or biological signals, and using a combination of multiple strategies are expected to further improve the ability of future conduits to promote the directional growth of nerves, making their effect in promoting the repair of injured nerves one step closer to autologous transplantation.

### Current limitations and challenges

#### Hindrances to clinical translation of the strategies

Although there have been several NGCs receiving clinical approval for nerve repair, they can only be used to repair the short-mid defects within 3 cm due to their limited ability to promote nerve regeneration [10, 11]. Many explorations as researchers have made to improve the conduits, there are still lots of limitations and challenges associated with enhancing the directional guidance capability of them.

In terms of structural improvement, no matter which directional structure among channels, microgrooves, micropores and microfibers is fabricated within the conduits, it remains difficult to imitate the fascicular structure of natural nerves. In terms of substance loading, rapid degradation of the loaded substances and their leakage from NGCs may prevent them from functioning for an adequate time. Some of the substances may also be immunogenic or cytotoxic, so the safety of loading them within NGCs still needs to be further elucidated. Moreover, to fabricate ideal NGCs, there exist many other characteristics that should be considered, including their mechanical properties, permeability, biodegradability, biocompatibility and ease of surgical handling [70]. These obstacles make it difficult to translate the above-mentioned strategies from bench to bedside, and the researchers seem to encounter bottlenecks when they attempt to further improve the conduits.

#### Possible future research directions

To break the bottlenecks encountered in current studies, here are several possible future research directions. In view of the problem that the directional structures within existing NGCs are quite different from the ones of nerves, it may be possible to use 3D printing to customize the fascicular structure within NGCs based on the position and shape of the fascicles in the injured nerves, then each fascicle of the nerve stumps can be accurately aligned with the custom-made structure within the conduits to achieve individualized repair. As for solving the problem of rapid failure of the substances loaded within NGCs, future researches can focus on the development of sustained-release systems for them.

In addition to the above-mentioned guidance-based strategies, future researches can also try applying some non-guidance-dependent ones at the same time. For example, when using NGCs to repair nerve defects, local or systemic use of immunosuppressants is also a strategy that may promote nerve regeneration. In the studies on nerve repair, the immunosuppressants widely used to reduce tissue rejection are calcineurin inhibitors such as cyclosporine A and tacrolimus [71–73], which inhibit T lymphocytes by reducing the release of interleukin-2 (IL-2). Furthermore, antimetabolites (such as mycophenolate mofetil and azathioprine) [72, 74] as well as glucocorticoids (such as prednisolone and dexamethasone) [72, 74, 75] have also been used as immunosuppressants in related researches. Besides, strategies providing a favorable microenvironment to promote neural self-organization may also be used to promote nerve regeneration. Some studies have shown that covalently immobilized interferon-*γ* (IFN-*γ*) can promote neural stem cells to form rosettes through neural self-organization, and can further develop into neural tubes and neuroepithelium [76]. Application of these strategies may complement the guidance-based approaches to promote overall directional nerve growth.

## Data Availability

No datasets were generated or analyzed during the current study.

## Abbreviations

NGCs: Nerve guidance conduits
PNI: Peripheral nerve injury
SCs: Schwann cells
M2: Pro-regenerative macrophage
M1: Pro-inflammatory macrophage
IL-4R*α*: Interleukin-4 receptor subunit alpha
TLR-4: Toll-like receptor 4
IL-4: Interleukin-4
PKB/Akt: Protein kinase B or Akt
eNOS: Endothelial nitric oxide synthase
VEGF: Vascular endothelial growth factor
NGF: Nerve growth factor
p75^NTR^: 75 KD neurotrophin receptor
AMPK: AMP-activated protein kinase
mTOR: Mammalian target of the rapamycin
PVDF: Poly-vinylidene fluoride
PEDOT: Poly-(3,4-ethylenedioxythiophene)
PPy: Poly-pyrrole
PANI: Poly-aniline
GO: Graphene oxide
rGO: Reduced graphene oxide
IL-2: Interleukin-2
IFN-*γ*: Interferon-*γ*
